# Prognostic Significance of Erythropoietin in Pancreatic Adenocarcinoma

**DOI:** 10.1371/journal.pone.0023151

**Published:** 2011-08-01

**Authors:** Thilo Welsch, Stefanie Zschäbitz, Verena Becker, Thomas Giese, Frank Bergmann, Ulf Hinz, Shereen Keleg, Anette Heller, Bence Sipos, Ursula Klingmüller, Markus W. Büchler, Jens Werner, Nathalia A. Giese

**Affiliations:** 1 Department of General, Visceral and Transplantation Surgery, University Hospital Heidelberg, Heidelberg, Germany; 2 Division Systems Biology of Signal Transduction, German Cancer Research Center and Bioquant, Heidelberg University, Heidelberg, Germany; 3 Institute of Immunology, University Hospital Heidelberg, Heidelberg, Germany; 4 Institute of Pathology, University Hospital Heidelberg, Heidelberg, Germany; 5 Institute of Pathology, University Hospital Tübingen, Tübingen, Germany; University of Nebraska Medical Center, United States of America

## Abstract

**Background:**

Erythropoietin (Epo) administration has been reported to have tumor-promoting effects in anemic cancer patients. We investigated the prognostic impact of endogenous Epo in patients with pancreatic ductal adenocarcinoma (PDAC).

**Methodology:**

The clinico-pathological relevance of hemoglobin (Hb, n = 150), serum Epo (sEpo, n = 87) and tissue expression of Epo/Epo receptor (EpoR, n = 104) was analyzed in patients with PDAC. Epo/EpoR expression, signaling, growth, invasion and chemoresistance were studied in Epo-exposed PDAC cell lines.

**Results:**

Compared to donors, median preoperative Hb levels were reduced by 15% in both chronic pancreatitis (CP, p<0.05) and PDAC (p<0.001), reaching anemic grade in one third of patients. While inversely correlating to Hb (*r* = −0.46), 95% of sEPO values lay within the normal range. The individual levels of compensation were adequate in CP (observed to predicted ratio, O/P = 0.99) but not in PDAC (O/P = 0.85). Strikingly, lower sEPO values yielding inadequate Epo responses were prominent in non-metastatic M0-patients, whereas these parameters were restored in metastatic M1-group (8 vs. 13 mU/mL; O/P = 0.82 vs. 0.96; p<0.01)—although Hb levels and the prevalence of anemia were comparable. Higher sEpo values (upper quartile ≥16 mU/ml) were not significantly different in M0 (20%) and M1 (30%) groups, but were an independent prognostic factor for shorter survival (HR 2.20, 10 vs. 17 months, p<0.05). The pattern of Epo expression in pancreas and liver suggested ectopic release of Epo by capillaries/*vasa vasorum* and hepatocytes, regulated by but not emanating from tumor cells. Epo could initiate PI3K/Akt signaling via EpoR in PDAC cells but failed to alter their functions, probably due to co-expression of the soluble EpoR isoform, known to antagonize Epo.

**Conclusion/Significance:**

Higher sEPO levels counteract anemia but worsen outcome in PDAC patients. Further trials are required to clarify how overcoming a sEPO threshold ≥16 mU/ml by endogenous or exogenous means may predispose to or promote metastatic progression.

## Introduction

Anemia in cancer patients is a common symptom caused either by the tumor itself or by cytotoxic treatment [1]. In response to decreased hemoglobin (Hb) levels, the erythropoiesis-stimulating agent erythropoietin (Epo) is produced in the kidney and subsequently triggers erythropoiesis in the bone marrow and the release of erythrocytes into the blood circulation, thus restoring the Hb level. In cancer, this feedback mechanism seems to be frequently disrupted, yielding an inadequate Epo response [2], [3]. Administration of exogenous (recombinant human) rhEpo has been approved for the correction of chemotherapy-induced anemia in patients with non-hematopoietic malignancies, leading to a reduction in blood transfusion requirements and an improvement in quality of life [4]–[6]. According to current guidelines, rhEpo can be administered to patients when dosed to a target Hb level of less than 12 g/dl [7], [8]. A meta-analysis revealed no negative effect on tumor progression if rhEpo was used in accordance with those guidelines [9]. Still, several clinical trials have shown a higher risk of thrombovascular events, decreased survival, and worse tumor control, calling into question the safety and benefit of rhEpo treatment in patients with solid tumors [8]–[10]. As summarized in a recent report, only one out of 19 clinical trials showed a positive impact of rhEpo on overall survival (hazard ratio 1.3), whereas 10 studies did not demonstrate any effect and 8 trials demonstrated worse survival for the Epo arm [11].

Those data also turned attention to the role of endogenous Epo in carcinogenesis [12]–[16]. Several studies have attempted to explore direct effects of Epo on tumor cells and the possible mechanisms for Epo-mediated tumor progression, but the data are still controversial. Epo is a 30.4-kDa protein whose binding to the transmembrane Epo receptor (EpoR) initiates signaling through several transduction cascades: Jak2/STAT5, PI3K/Akt, ERK1/2, phospholipase C and D, and NF-κB [17]–[20]. These pathways appear to transduce Epo/EpoR signals not only in erythroid precursors but also in malignant cells [12], [14], [21], [22]. The published data reporting an Epo-mediated impact on signaling, proliferation, survival or invasion varied greatly with different tumor cells and respective experimental conditions. The controversy over the functionality of EpoR in malignant cells was heightened by the discovery that cells may express multiple EpoR isoforms, with only a very small fraction being at the cell surface [23]–[25]. Importantly, the finding that the widely used anti-EpoR antibody C-20 cross-reacts with heat-shock protein 70 (HSP70) called into question C-20-based findings of EpoR in tumor cells and tissues [26], [27].

Pancreatic ductal adenocarcinoma (PDAC) is one of the most aggressive and deadliest malignancies, and also requires the highest rate of transfusions among cancer patients undergoing cytotoxic therapies [28]–[30]. Chemotherapy is standard in the adjuvant and palliative settings, and aggravates anemia. Considering the reported negative effects of rhEpo treatment, the use of rhEpo to correct anemia in PDAC patients should be carefully assessed. In the present study, we hypothesized that the level of endogenous Epo might be a risk factor for PDAC progression in both anemic and non-anemic patients, and therefore investigated whether and how the individual Epo response can determine the degree of cancer aggressiveness in PDAC patients. The expression of Epo/EpoR in blood and tissue samples was analyzed in the context of clinico-pathological parameters in donors, chronic pancreatitis (CP) patients, and PDAC patients. The possibility of direct pro-malignant effects and increased chemoresistance was assessed in PDAC cells exposed to rhEpo.

## Materials and Methods

### Patients and specimens

The study was conducted in accordance with the Helsinki Declaration; specimen collection was approved by the ethical committee of the University of Heidelberg (votes 301/2001 and 159/2002) and written informed consent was obtained from the patients. The study was performed with tissue and blood samples obtained from patients admitted to the Department of General, Visceral and Transplantation Surgery, University Hospital Heidelberg [30], between February 2002 and February 2005 for surgical treatment of PDAC (n = 150) or chronic pancreatitis (CP, n = 42). Diagnoses were established by a pathologist according to the World Health Organization (WHO) classification. Clinical stages are given as defined by the Union for International Cancer Control (AJCC/UICC; 7^th^ Edition, 2009). The normal pancreatic specimens (n = 29) were collected during donor organ procurement if no suitable recipient had been found for the donor's pancreas through the Eurotransplant program. The general characteristics of the patients are given in Table 1: the blood specimens were processed to measure levels of Hb and Epo, whereas tissues were used for analyses of Epo/EpoR mRNA and protein expression. The exact number of analyzed specimens in each study group is also shown in the figure legends.

**Table 1:** Patients information.

A. General characteristics of the groups								
Groups	No. of patients	Age [years]Median (IQR)	Hb [g/dl]Median (IQR)	Serum Epo [mU/ml]Median (IQR)	O/P ratioMean±SD	Epo mRNA [copies/10 kCPB]Median (IQR)	EpoR mRNA [copies/10 kCPB]Median (IQR)	Survival [months]Median
**Donor**	38	50.5 (34.0–59.8)	15.1 (13.9–16.3) n = 9	12.7 (8.7–13.3) n = 9	1.01±0.12	20 (13–25) n = 29	174 (124–281) n = 29	
Male	25	46.0 (30.5–60.5)	15.9 (14.9–16.3) n = 6	12.9 (11.3–13.7) n = 6	1.06±0.10	16 (10–25) n = 19	148 (104–255) n = 19)	
Female	13	52.0 (43.5–60.5)	13.8 (13.5–13.9) n = 3	9.2 (8.1–12.7) n = 3	0.90±0.08	22 (15–26) n = 10	240 (152–544) n = 10)	
**CP**	42	47.0 (38.0–54.0)	12.9 (12.0–14.2) n = 42	12.0 (8.9–18.8) n = 13	0.99±0.23	7 (2–13) n = 29	351 (219–533) n = 29)	
Male	31	50.0 (37.0–56.0)	13.6 (12.3–14.5) n = 31	11.3 (8.0–15.1) n = 10	0.92±0.17	9 (2–16) n = 21	355 (219–533) n = 21)	
Female	11	44.0 (40.0–48.0)	11.8 (10.8–12.9) n = 11	18.2 (13.7–74.4) n = 3	1.25±0.25	5 (2–9) n = 8)	322 (218–538) n = 8)	
**PDAC**	150	65.0 (56.0–70.3)	13.0 (11.9–13.9) n = 150	9.8 (5.6–15.2) n = 87	0.85+0.24	1 (0–2) n = 104	176 (94–302) n = 104)	18.0
Male	80	63.5 (56.0–70.8)	13.7 (12.0–14.5) n = 80	8.5 (5.3–15.5) n = 42	0.87±0.25	0 (0–4) n = 60	149 (95–281) n = 60)	17.0
Female	70	65.0 (57.8–70.3)	12.6 (11.8–13.5) n = 70	10.0 (6.0–15.3) n = 45	0.84±0.24	1 (0–2) n = 44	183 (94–418) n = 44)	18.5

**B. PDAC sub-cohorts**								
**Study cohort**		**PDAC**	**Stage I/IIA**	**Stage IIB**	**Stage III**	**Stage IV**	**Anemic**	**Non-anemic**
**Total**	No. of patients	150	25	85	9	31	43	107
	Age [years; median (IQR)]	65 (56–70)	62 (55–73)	66 (59–71)	58 (56–65)	64 (58–70)	65 (57–73)	65 (56–70)
	Hb [g/dL; median (IQR)]	13.0 (11.9–13.9)	12.9 (12.3–13.9)	13.2 (12.0–14.2)	12.6 (10.3–13.2)	12.8 (11.5–13.9)	11.4 (10.3–11.8)	13.6 (12.8–14.4)
	Anemic	29%	20%	27%	44%	35%		
	Survival [months; median]	18.0	28.5	18.0	17.5	10.0	11.0	19.0
**Serum**	No. of patients	87	16	44	4	23	31	56
	Age [years; median (IQR)]	65 (58–70)	62 (55–72)	66 (60–70)	62 (57–66)	64 (58–71)	64 (58–72)	66 (57–70)
	Hb [g/dL; median (IQR)]	12.8 (11.8–13.9)	12.9 (11.9–13.9)	12.9 (11.8–14.1)	12.4 (10.6–13.3)	12.3 (11.5–13.7)	11.5 (10.7–11.8)	13.7 (13.9–14.4)
	Anemic	36%	25%	34%	30%	43%		
	sEpo [mU/mL; median (IQR)]	9.8 (5.6–15.2)	9.1 (4.6–11.6)	7.3 (5.0–15.5)	8.8 (5.4–10.9)	12.5 (8.7–18.4)	14.9 (8.4–22.2)	7.5 (4.9–11.0)
	O/P [mean±SD]	0.85±0.24	0.79±0.21	0.82±0.26	0.77±0.16	0.96±0.22	0.95±0.24	0.80±0.23
	Survival [months; median]	17.0	32.0	18.0	9.0	8.5	13.5	18.0
**Tissue**	No. of patients	104	19	68	5	12	24	80
	Age [years; median (IQR)]	65 (56–71)	62 (54–73)	66 (58–71)	56 (55–64)	61(55–67)	67 (57–76)	64 (56–70)
	Hb [g/dL; median (IQR)]	13.3 (12.2–14.2)	12.9 (12.2–13.7)	13.3 (12.0–14.3)	12.6 (10.2–14.6)	13.9 (13.0–14.4)	11.5 (10.2–11.9)	13.7 (12.9–14.5)
	Anemic	23%	21%	25%	40%	8%		
	sEpo [mU/mL; median+IQR]	7.0 (4.5–10.0) n = 41	9.1 (4.2–16.3) n = 10	6.1 (4.6–9.9) n = 27	n/a	7.6 (3.8–8.7) n = 4	9.2 (4.7–23.6) n = 12	6.5 (4.5–9.1) n = 29
	Survival [months; median]	22.0	30.0	18.0	30.0	12.0	13.0	23.0

### Hematological analyses

Hb values were determined in pre-operative blood samples of PDAC (n = 150) and CP (n = 42) patients and used to diagnose anemia as defined by the European Organization for the Research and Treatment of Cancer (EORTC) at Hb levels ≤12 g/dl. Epo levels in sera of PDAC (n = 87) and CP (n = 13) sub-cohorts as well as healthy volunteers (n = 9) were analyzed using the IMMULITE® detection system and Epo kit according to the manufacturer's instructions (DPC Biermann GmbH, Bad Nauheim, Germany).

The adequacy of Epo response to a given degree of anemia was defined by a standard method calculating observed to predicted sEPO ratio (O/P) [3], [31]–[34]. The regression equation log(Epo) = 1.666−(0.04147×Hb) was established with sEpo and Hb values from 22 non-cancerous reference subjects (donors and CP patients) and then used to predict Hb-adequate Epo values in the individuals with PDAC. The mean O/P ratio in reference subjects was 1.00±0.19 (95% confidence interval: 0.92–1.09).

### Cell cultures and Epo treatment

The authenticity of established pancreatic cell lines AsPC-1, BxPC3, Colo357, Capan-1, MiaPaCa-2, PANC-1, Su8686, and T3M4 was certified by DSMZ (German Collection of Microorganisms and Cell Cultures, GmbH, Braunschweig, Germany). The cells were cultured under hypoxic (0.75% O_2_+10% CO_2_; in a chamber produced by Billups-Rothenberg, Del Mar, CA) or normoxic (21% O_2_+5% CO_2_) conditions in RPMI-1640 (PAA, Cölbe, Germany) supplemented with 0–10% of fetal bovine serum (FBS, PAN Biotech, Aidenbach, Germany). The IL-3-dependent pro-B cell line Ba/F3 or the fibroblast cell line NIH/3T3 were stably transduced with either pMOWS-hEpoR or the empty vector (mock-transduction) and served as a positive or negative control, respectively. Ba/F3 cells were maintained with 10% of WEHI-3B supernatant as a source of IL-3. [20].

The cells were exposed to rhEpo (Erypo®, Ortho Biotech, Janssen-Cilag GmbH, Neuss, Germany) at final concentrations of 1–50 U/ml. Depending on the type of experiment, the cells were harvested at different time points, as indicated in the main text. The signal transduction studies included pretreatment of cells with the PI3K-inhibitor LY2940002 (Sigma, Deisenhofen, Germany) at a final concentration of 30 µM added 30 min prior to the experiments or addition of the monoclonal anti-EpoR antibody (MAB307, Table 3) at 30 µg/ml as previously described to antagonize binding of Epo to the EpoR [16], [20].

### Analysis of Epo and EpoR mRNA expression

mRNA was isolated from tissues and cells, converted to cDNA, and amplified by PCR (real-time quantitative qRT-PCR) using kits and automated equipment from Roche Applied Sciences (RAS, Mannheim, Germany) as described previously [35]. The number of Epo and EpoR transcripts in the samples measured in the LightCycler® (RAS) with commercially available kits (Search-LC, Heidelberg, Germany) was normalized to the expression of the housekeeping gene cyclophilin B. The final data are presented as number of Epo or EpoR transcripts per 10,000 CPB copies (10 kCPB).

To check the expression of EpoR isoforms, PCR was performed using primers previously published by Arcasoy et al., as listed in Table 2 [24]. PCR products were separated by agarose gel electrophoresis and visualized by staining the gel with ethidium bromide.

**Table 2:** Overview of EpoR RT-PCR primers [24].

Primer	EpoR gene location	Sequence	Product size (bp)	Isoform
EpoR_FL1	Exon 8 sense	GCTCCCAGCTCTTGCGTCCA	316	Full length EpoR
EpoR_FL2	Non-coding antisense	TCGCCATCCCTGTTCCATAA		
EpoR_FL3	Exon 7 sense	ATCCCGAGCCCAGAGAGCGAGT	137	Full length EpoR
EpoR_FL4	Exon 8 antisense	AGGGAAGCAGGTGGGTCCTCCGTG		
EpoR_S5	Intron 5 sense	GGAGCCAGGGCGAATCACGG	204	Sol-EpoR (Intron 5 unspliced)
EpoR_S6	Exon 7 antisense	GCCTTCAAACTCGCTCTCTG		

### Testing of anti-EpoR antibodies

Recent studies revealed a lack of specificity for C-20 and other anti-EpoR antibodies due to cross-reactivity with HSP70 [26]. In the present study we tested the ability of 5 different antibodies (Table 3) to detect EpoR by immunoprecipitation, Western blot, FACS and immunohistochemistry. To differentiate between specific and non-specific binding, we used i) hEpoR- or mock-transduced BaF/3 and NIH/3T3 cells, ii) bone marrow specimens and iii) soluble EpoR (sol-EpoR) for blocking set-ups. As a result, the monoclonal 3D10 antibody recognizing an extracellular domain of EpoR (Sigma) was chosen for the detection of EpoR in different assays (except flow cytometry).

**Table 3:** Primary antibodies.

Antigen	Antibody type	Application* and Dilution	Manufacturer
hEpoR (C-20, cytoplasmic domain)	Rabbit polyclonal	IB, 1∶2000–1∶5000	Santa Cruz Biotechnologies, Heidelberg, Germany
hEpoR (M-20, cytoplasmic domain)	Rabbit polyclonal	IB, 1∶500; IP, 1∶250	Santa Cruz Biotechnologies, Heidelberg, Germany
hEpoR (clone 38409, extracellular domain)	Mouse monoclonal	FC, 10 µL/2×10^5^ cells	R&D Systems, Minneapolis, MN, USA
Mouse IgG2b Isotype control	Mouse monoclonal	FC, 20 µL/2×10^5^ cells	R&D Systems, Minneapolis, MN, USA
hEpoR (clone MAB307, extracellular domain)	Mouse monoclonal	BA, 30 µg/µl	R&D Systems, Minneapolis, MN, USA
hEpoR (3D10, extracellular domain)	Mouse monoclonal	IF, 1∶25; IB, 1∶500; IHC, 1∶100; IP, 3 µL	Sigma, Deisenhofen, Germany
Epo (n-19)	Goat polyclonal	IHC 1∶50	Santa Cruz Biotechnologies, Heidelberg, Germany
Akt	Rabbit polyclonal	IB, 1∶1000	Cell Signaling Technology, Inc., Danvers, MA, USA
Phospho-Akt (ser473)	Rabbit polyclonal	IB, 1∶1000	Cell Signaling Technology, Inc., Danvers, MA,USA
STAT5 (C-17)	Rabbit polyclonal	IP, 5 µL; IB, 1∶1000	Santa Cruz Biotechnologies, Heidelberg, Germany
Phospho-STAT5 (Tyr 694)	Rabbit polyclonal	IB, 1∶1000	Cell Signaling Technology, Inc., Danvers, MA,USA

*BA, blocking antibody; IB, immunoblot; IF, immunofluorescence; IHC, immunohistochemistry; IP, immunoprecipitation; FC, flow cytometry.

### Immunohistochemistry

Immunohistochemistry was performed as previously described [36]. Briefly, 4 µm-thin paraffin-embedded tissue sections were deparaffinized and rehydrated in progressively decreasing concentrations of ethanol. After retrieval of antigens by boiling in 10 mM citrate buffer, the sections were subsequently exposed to 3% H_2_O_2_ in methanol, universal blocking solution (Powerblock; Biogenix, San Ramon, CA, USA), and the goat anti-hEpo or mouse anti-hEpoR primary antibodies (Table 3). Sections incubated with goat IgG or mouse IgG2b (Dako, Glostrup, Denmark) served as negative isotype controls for n-19 and 3D10 antibodies. The anti-Epo antibody was validated by showing immunopositivity of the tubular epithelial cells in the kidney (not shown). For EpoR, the specificity of the staining was additionally controlled by incubation of the sections with the anti-EpoR antibody pre-treated with a 5-fold excess of soluble EpoR (sol-EpoR). After overnight incubation at 4°C, the secondary horseradish peroxidase (HRP)-labeled antibodies were applied at room temperature for 45 min (donkey anti-goat IgG for EPO; Santa Cruz Biotechnology/SCBT, Heidelberg, Germany, or polymer-conjugated goat anti-mouse IgG for EpoR; EnVision™^+^System, Dako). The HRP-reaction product was visualized using a DAB/H_2_O_2_ substrate mixture kit (EnVision™, Dako) and sections were counterstained with Mayer's hematoxylin.

### Immunoprecipitation (IP) and Western blot

Cells were lysed in 1% Nonidet P-40 lysis buffer (1% NP-40, 150 mM NaCl, 20 mM Tris at pH 7.4, 10 mM NaF, 1 mM ZnCl_2_ at pH 4.0, 1 mM EDTA at pH 8.0, 1 mM MgCl_2_, 10% glycerol, 1 mM Na_3_VO_4_) supplemented with proteinase inhibitor cocktail (Roche) [16], [20]. For IP, the lysates were incubated 20 minutes on ice, cleared by centrifugation, and incubated with a primary antibody (table 3) and protein A-sepharose beads at 4°C overnight. The immunocomplexes were washed with lysis buffer and TNE buffer (10 mM Tris at pH 7.4, 100 mM NaCl, 1 mM EDTA at pH 8.0, 100 µM Na_3_VO_4_). Protein samples were denatured at 95°C for 2 min, separated using a 10% polyacrylamide SDS gel. For non-IP Western blot analysis, 40 µg of protein from cell lysates or 2 µl of sol-EpoR (50 µg/ml, Sigma) were separated using 4–12% gel (NuPAGE, Invitrogen GmbH, Darmstadt, Germany). Upon transfer to a nitrocellulose membrane (BioRad, Munich, Germany), the blotted samples were blocked with 5% SlimFast powder (Allpharm, Messel, Germany) in TBS buffer with 0.05% Tween-20, exposed to primary antibodies (Table 3) overnight at 4°C, and incubated with corresponding anti-rabbit or anti-mouse HRP-conjugated secondary antibodies (1∶2,000; Santa Cruz, Heidelberg, Germany) for 1 h at room temperature. The signal was visualized using Amersham ECL detection reagents (GE Healthcare, Buckinghamshire, UK).

### Flow cytometry

Cells were resuspended in FACS buffer (PBS, 2% FBS, 0.01% sodium azide), blocked with FCR Blocking Reagent/human (Miltenyi Biotech GmbH, Bergisch Gladbach, Germany) and incubated with FITC-labelled anti-EpoR antibody (clone 38409, extracellular domain) or IgG2b isotype control immunoglobulin (both R&D Systems, Minneapolis, MN, USA, table 3). FACS analysis was performed with the FACS LSR-II system (BD Biosciences, Heidelberg, Germany).

### Functional assays (cell invasion, growth and chemoresistance)

To evaluate cell invasion, upper chambers of Biocoat Matrigel-TM invasion chambers (8 µm pore size/PET membrane, BD) were seeded with serum-starved (24 h) cells. The bottom chamber contained RPMI-1640 with Epo at 1–50 U/ml. After 24 h, the membranes with invaded cells were fixed in 70% ethanol and stained with Mayer's hemalum solution (Merck KGaA, Darmstadt, Germany). The cells were counted in 3 visual fields under 100-fold magnification.

To evaluate growth and chemoresistance, a 3-(4,5-dimethylthiazol-2-yl)-2,5-diphenyltetrazolium bromide (MTT) test was performed. The cells were seeded in 96-well plates and incubated with various concentrations of Epo (1–50 U/ml), with or without the chemotherapeutic agents 5-fluorouracil (5-FU) (Sigma) and gemcitabine (Fresenius, Bad Homburg, Germany) at 0.1–10 µM. MTT reagent was added after 48 h of incubation, and the accumulation of formazan in live cells was determined upon solubilisation of the product and measuring optical density at 570 nm. All assays were performed in triplicate.

### Statistical analyses

Data were analyzed with GraphPad Prism (GraphPad Software, La Jolla, CA) and SAS (version 9.1; SAS Institute, Cary, NC, USA). The quantitative variables Hb, sEpo, tissue Epo/EpoR mRNA are presented as dot blots or box and whisker plots displaying the median, interquartile range (IQR), and the 5^th^ and 95^th^ percentiles. Significance of differences between groups was assessed by the nonparametric Mann-Whitney U test (to compare 2 groups) or the Kruskal-Wallis with Dunn's tests (to compare 3 and more groups). Linear correlation analyses were performed using the Spearman test. Multivariate Cox regression analysis was performed to identify independent prognostic markers among parameters given in Table 1. To analyze the impact of Epo levels on the overall survival, the corresponding quartiles were used to divide patients into groups. The overall survival from the date of initial surgery was estimated using the Kaplan-Meier method. The observations on 14 PDAC patients still alive at the end of the follow-up period in October 2010 were censored. Differences between survival curves were analyzed using the log-rank test. For all assays, two-sided p values were computed and a difference was considered statistically significant at p≤0.05 (*), p<0.01(**) or p<0.001(***).

## Results

### Higher levels of circulating Epo are associated with a worse prognosis in PDAC

**Figure 1:**
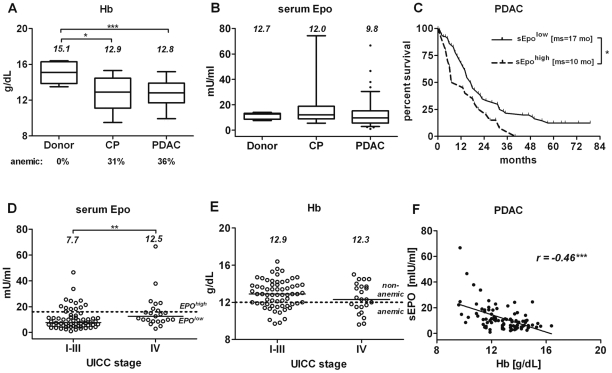
Level of sEpo but not Hb was associated with a worse prognosis in PDAC. The differences in preoperative levels of Hb (A) and sEpo (B) in blood of donors (n = 9), CP (n = 13) and PDAC (n = 87) patients (see table 1A and 1B for full information regarding studied sub-cohorts). (C) Kaplan-Meier analysis of survival data showing that a higher level of sEpo (upper quartile in panel B, ≥16 mU/ml) was associated with shorter survival of PDAC patients; *ms* = median survival time, in months. (D) Reduced sEpo levels in stage I–III PDAC patients (n = 64) and their restoration in metastatic patients (n = 23). (E) Prevalence of anemia and Hb values were equally distributed during PDAC progression. (F) sEpo inversely correlated with Hb in PDAC. The numbers in panels A, B, D and E depict median levels. Statistically significant differences are labeled by asterisks: p≤0.05 (*), p<0.01(**) or p<0.001(***).

The median Hb level in patients with PDAC was 15% lower than in donors (p<0.001, Figure 1A and Table 1). Likewise, in patients with CP, median Hb level was significantly decreased (p<0.05). A third of patients with PDAC or CP were mildly anemic (≤12 g/dl) prior to surgical resection. 95% of the sEpo values in PDAC patients were within the known normal range (5–34 mU/ml) and did not differ significantly from those in donor and CP groups (median sEPO: 9.8 mU/ml vs. 12.7 and 12.0 mU/ml, respectively; p = 0.15; Figure 1B). Nevertheless, multivariate analysis revealed that the level of endogenous sEpo –but not Hb–was an independent negative prognostic factor for PDAC patients (hazard ratio, HR 2.20, p = 0.01). Patients with sEpo>16 mU/ml (sEpo^high^, upper quartile) had a significantly shorter median survival (10.0 vs. 16.7 months; p = 0.03) and increased 3-year mortality rates (100% vs. 75%, Figure 1C). The proportions of sEpo^high^ individuals in the subgroups with localized (M0, UICC stages I–III) and metastatic (M1, UICC stage IV) disease stages were comparable (M0 vs. M1: 20.3% *vs.* 30.4%; p = 0.39), even though tumor stage (but not grade, G3 vs. G1/2, p = 0.74) was another independent prognostic factor in PDAC (UICC I+IIa vs. IIb: HR 0.43, p = 0.030; III+IV vs. IIb: HR 2.09, p = 0.020).

Median sEpo level in patients with stage IV was approximately double that of stage I–III patients (12.5 and 7.7 mU/ml; p<0.01, Figure 1D) but comparable to donor and CP groups (Figure 1B). Lower Epo levels in stage I–III patients could be attributed either to the lack of anemia in this group or to an inadequate Epo response to a given degree of anemia. Yet Hb values (Figure 1E) and the prevalence of anemia were comparable between different stages (UICC I–III vs. IV: 33% vs. 43%, p = 0.45; Table 1). Despite an inverse correlation between sEPO and Hb in PDAC patients (Spearman *r* = −0.46, p<0.001; Figure 1F), the Epo response in PDAC patients was lower than expected (mean O/P ratio: 0.85±0.24; 95% confidence interval [CI]: 0.80–0.91). However, this impaired Epo response was caused primarily by lower sEpo level consequently leading to reduced O/P ratios in stage I–III patients (mean observed to predicted ratio (O/P) = 0.82±0.26, 95%CI 0.76–0.89) as contrasted by higher sEpo values yielding an adequate Epo responses in CP (O/P = 0.99±0.23, 95%CI 0.85–1.13; p<0.05) and PDAC/stage IV cohorts (OP = 0.96±0.22; 95%CI 0.89–1.08; p<0.01).

### Aberrant ectopic expression of Epo in pancreatic diseases

**Figure 2:**
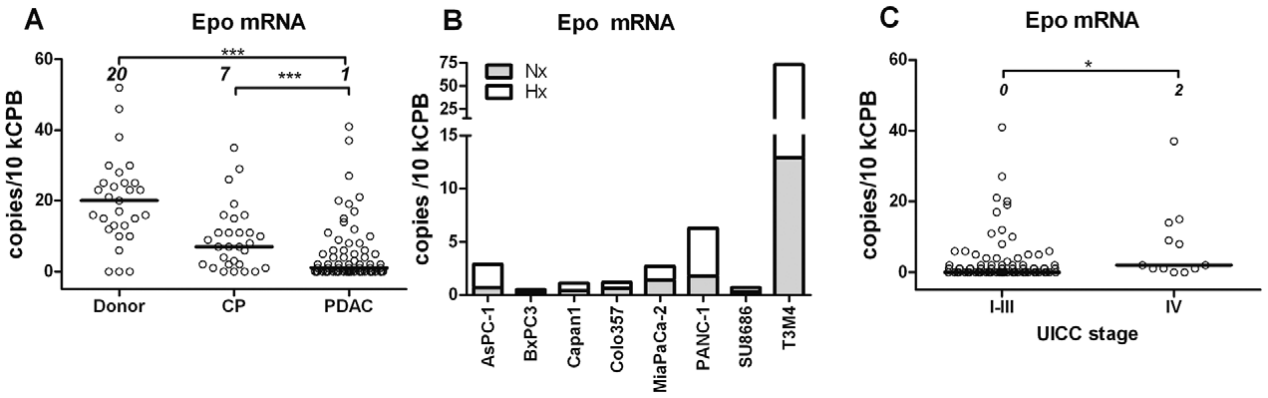
Aberrant expression of Epo mRNA in pancreatic tissues and tumor cells. (A) qRT-PCR analysis of pancreatic tissues revealed gradual loss of Epo mRNA expression in diseased pancreata. (B) Except for T3M4, low levels of Epo mRNA expression were found in pancreatic tumor cell lines exposed to or not exposed to 0.75%-hypoxia. *Nx* = normoxia; *Hx* = Hypoxia. (C) Barely detectable yet elevated Epo mRNA expression was found in pancreatic specimens obtained from stage IV PDAC patients (n = 12) compared to stage I–III (n = 92) patients. The numbers in panels A and C depict median levels. Statistically significant differences are labeled by asterisks: p≤0.05 (*), p<0.01(**) or p<0.001(***).

**Figure 3:**
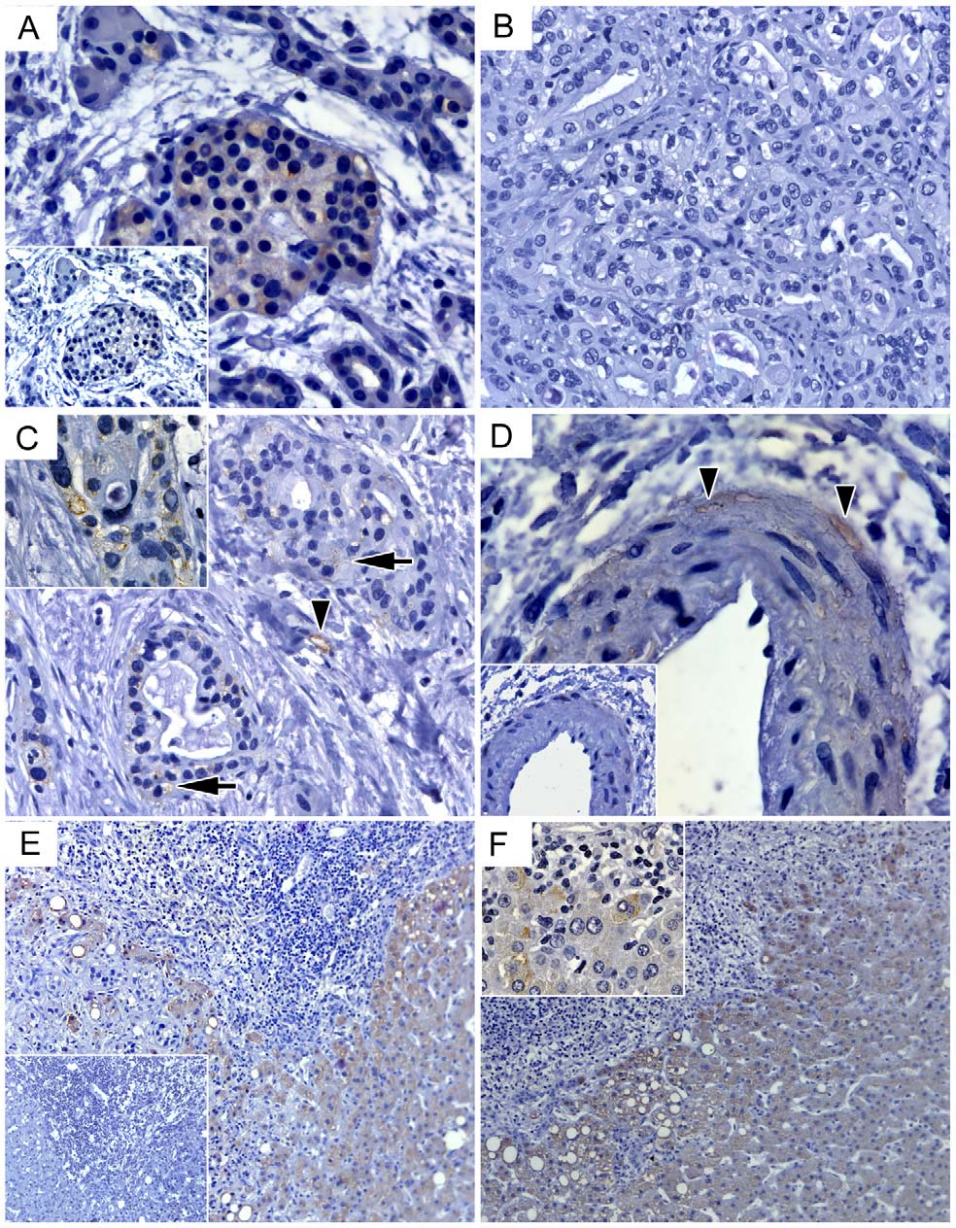
Ectopic sources of Epo in tissues of patients with pancreatic diseases. (A) Remnants of Epo-producing islets in degrading CP-parenchyma. (B) Epo-negativity of tumor cells in primary pancreatic lesion, except for sporadic focal staining observed in intracellular vacuoles of tumor cells (C, arrows and inset). Peripheral capillaries (C, arrowheads) and *vasa vasorum* (D, arrowheads) of bigger vessels frequently demonstrated Epo positivity in PDAC and CP. (E, F) In liver, cytoplasmic staining of hepatocytes was strong in areas directly bordering Epo-negative metastatic tumor cells and inflammatory infiltrates, but faded away distally, thus pointing to the spatially regulated *de novo* synthesis and creation of multiple Epo-rich niches. Insets in A, D and E depict negative (isotype IgG) controls; insets in C and F show high-magnification (×630) images of staining patterns in intracellular vacuoles of tumor cells and cytoplasm in hepatocytes.

The restoration of EPO response in PDAC/stage IV patients might reflect a ‘passive’ elevation of sEPO levels through ectopic (Hb- and kidney-independent) Epo release. Quantitative mRNA analysis confirmed the ability of the pancreas to produce Epo, but disclosed a progressive loss of Epo mRNA expression in CP by >70% and in PDAC by >95% (Figure 2A). This pattern suggested that Epo-producing islets [37] were disappearing together with the vanishing parenchyma (Figure 3A), and that accumulating PDAC cells were unable to compensate for islet-related losses. Indeed, immunohistochemical staining of PDAC samples showed that Epo-specific antibodies did not bind to the tumor cells (Figure 3B) - except for the rare foci which displayed Epo-positive intracellular vacuoles (Figure 3C). Furthermore, constitutive or hypoxia-stimulated Epo transcription was barely detectable in established PDAC cell lines, except in T3M4 cells showing robust Epo mRNA transcription and its 3-fold up-regulation under hypoxia (Figure 2B). This indicated that the PDAC cells were an unlikely source of ectopic Epo. In contrast, Epo-positive endothelium [21] was frequently found in peripheral capillaries and *vasa vasorum* of the bigger vessels in PDAC and also in CP (Figure 3D) but not in donor pancreata (not shown).

Although pancreatic tumor samples of stage IV patients showed statistically significant elevation of Epo mRNA levels compared to the non-metastatic group, the measured values were extremely low (Figure 2C). Furthermore, no significant correlation could be observed between pancreatic Epo mRNA and sEpo in PDAC patients (n = 41; Spearman *r* = −0.18, p = 0.26). Thus, we investigated whether a distant organ affected by metastasis could serve as source for ectopic Epo. Metastasized to the liver tumor cells rarely expressed Epo. However, Epo-negative metastases were frequently outlined by Epo-positive hepatic parenchyma featuring a remarkable gradient of the coloration that faded away in distal hepatocytes (Figure 3E–F). In non-metastatic parenchyma, the cytoplasmic Epo signal was detected in hepatocytes located near portal areas or inflammatory infiltrates (not shown). qRT-PCR analysis confirmed the ability of normal (25±15 copies/10 kCPB, n = 6 organ donor specimens) and metastatic (12±4 copies/10 kCPB, n = 12 biopsies) livers to express Epo. Thus, non-malignant hepatocytes emerged as a potential ectopic source of Epo, possibly being spatially controlled by the tumor cells.

### Variability of the EpoR expression in cancerous pancreata

Apparently, increased production, release, and delivery of Epo by non-malignant structures could create multiple Epo-rich niches. Their potential direct impact on tumor cells was assessed by studying i) the expression of EpoR in PDAC cells, ii) Epo-induced down-stream signaling, and iii) functional consequences of exposure to Epo.

**Figure 4:**
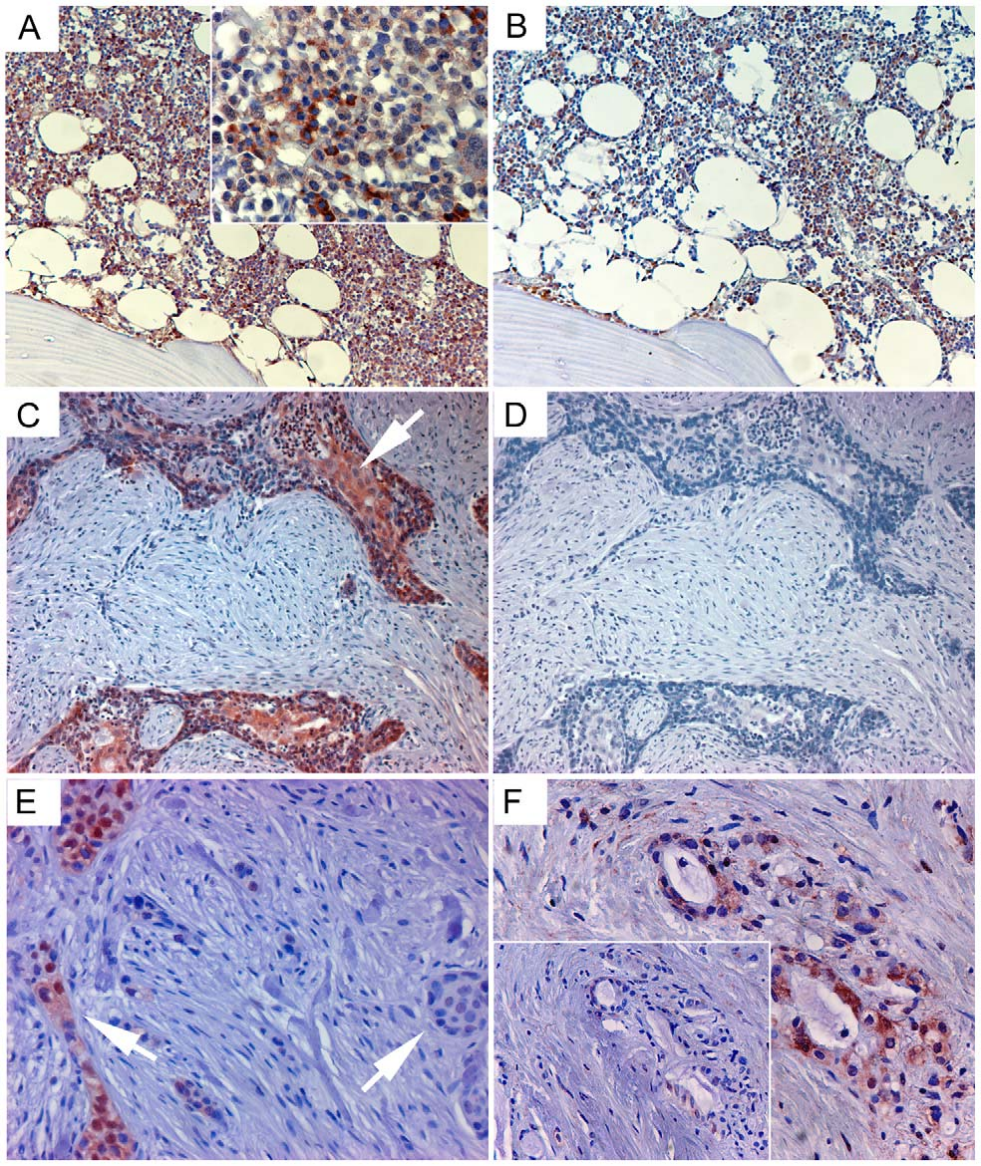
Detection of EpoR-positive tumor cells in PDAC pancreata. (A) Staining of erythroid precursor cells in human bone marrow with anti-EpoR 3D10 antibody (inset, ×400) and (B) loss of staining if the anti-EpoR antibody has been pre-incubated with soluble EpoR (sol-EpoR). (C, E) Varying intensity and focal character of EpoR-immunopositivity of tumor cells in PDAC tissues (arrows) and (D) blocking of EpoR signal with sol-EpoR. (F) Sporadic EpoR-immunopositivity of non-malignant structures within a PDAC sample and blocking of EpoR signal with sol-EpoR (inset).

In agreement with previous observations reporting that most of the EpoR was retained in the endoplasmic reticulum [38], we detected strong cytoplasmic staining of erythroid cells in bone marrow, with decrease of positivity after pre-incubation of antibody with sol-EpoR (Figure 4A–B). The staining of PDAC tissues revealed highly heterogeneous patterns of immunopositivity (Figure 4C–E). Whereas 37% (7/19) of specimens showed no staining, cytoplasmic signal was detected in the remaining cases, ranging from a weak sporadic to a strong abundant EpoR-positivity in 26% and 37% of cases, respectively. We could not identify characteristic distinctions between EpoR-positive and EpoR-negative cells (e.g., specific localization within the tumor or near vessels), nor was there a correlation with lymph node-positive N1 status of tumor or grade of tumor differentiation. In addition, few non-malignant structures within PDAC tissue samples also demonstrated EpoR immunopositivity (Figure 4F). The tissue expression of EpoR in normal pancreas and PDAC tissues was further confirmed through mRNA analysis (Table 1).

**Figure 5:**
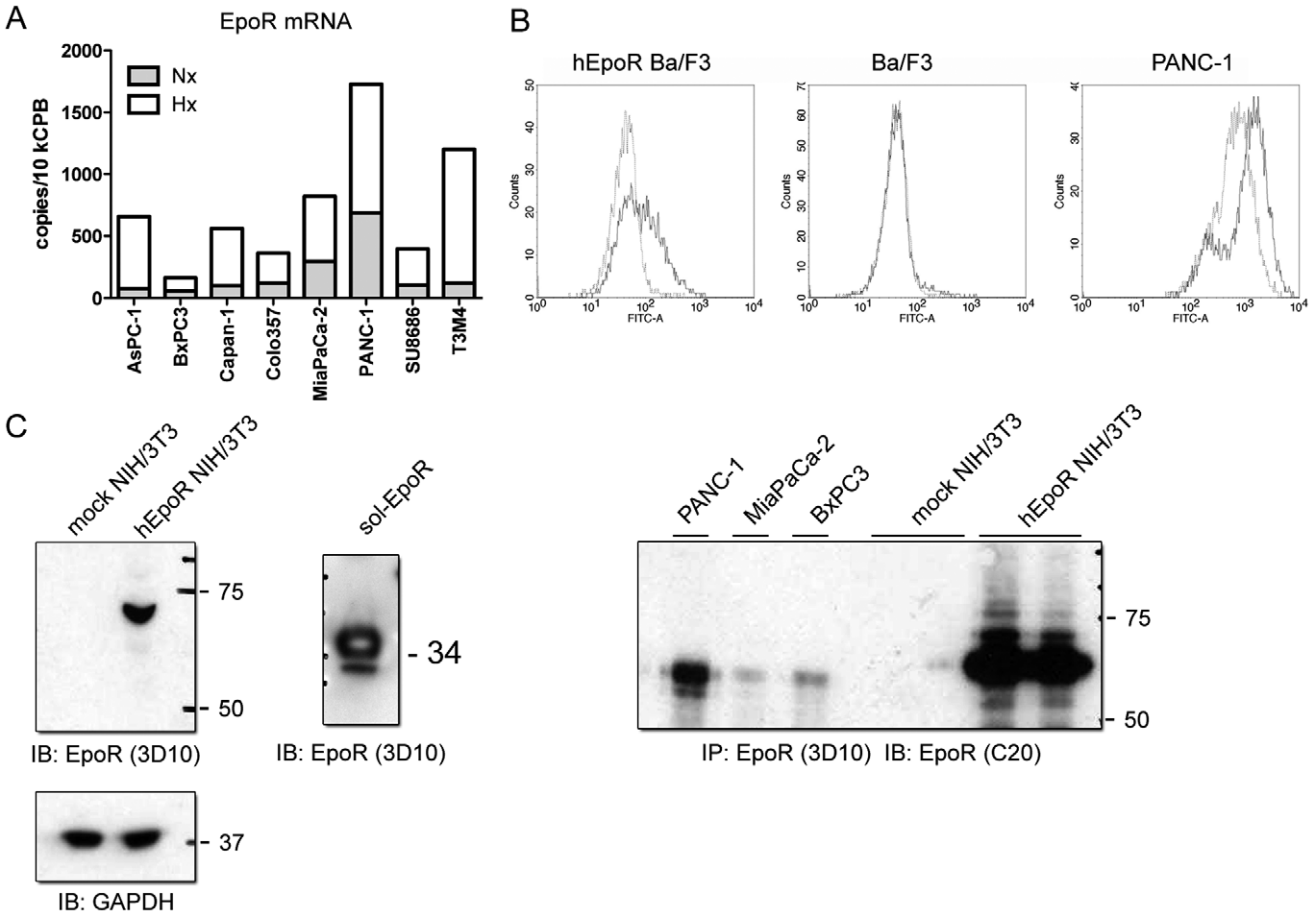
Expression of EpoR in PDAC cells. (A) qRT-PCR analysis revealed constitutive EpoR mRNA expression in all studied cancer cell lines and its strong up-regulation under hypoxic conditions. *Nx* = normoxia, *Hx* = hypoxia. (B) EpoR was detectable on the surface of hEpoR-transduced Ba/F3 cells and PANC-1 cells by flow cytometry analysis using an FITC-labeled antibody. Mouse IgG2b was used as a negative control. (C) Left panel: detection of the full-length EpoR protein in cell lysates of hEpoR-transduced NIH/3T3 cells and the recombinant sol-EpoR protein using the 3D10-antibody by Western blot analysis (with GAPDH as loading control). Right panel: detection of EpoR in pancreatic tumor cells and hEpoR-NIH/3T3 by immunoprecipitation with the 3D10-antibody followed by immunoblotting with the C-20 antibody.

The variability of EpoR expression was also observed in established pancreatic tumor cell lines. All eight studied cell lines expressed EpoR mRNA and showed a 2- to 10-fold up-regulation in hypoxic conditions (Figure 5A). The level of EpoR expression under normoxia ranged from 10 copies/10 kCPB in BxPC3 cells to over 300 copies/10 kCPB in PANC-1 cells. PANC-1 was the only cell line in which EpoR protein was detectable at the cell surface by flow cytometry using anti-EpoR antibodies (Figure 5B), whereas immunoprecipitation with the 3D10 antibody followed by immunoblotting with the C-20 antibody also allowed detection of EpoR protein in the cells with lower mRNA expression, such as MiaPaCa-2 and BxPC3 (Figure 5C).

### Epo activates PI3K/Akt but not Jak2/STAT5 signaling in PDAC cells

**Figure 6:**
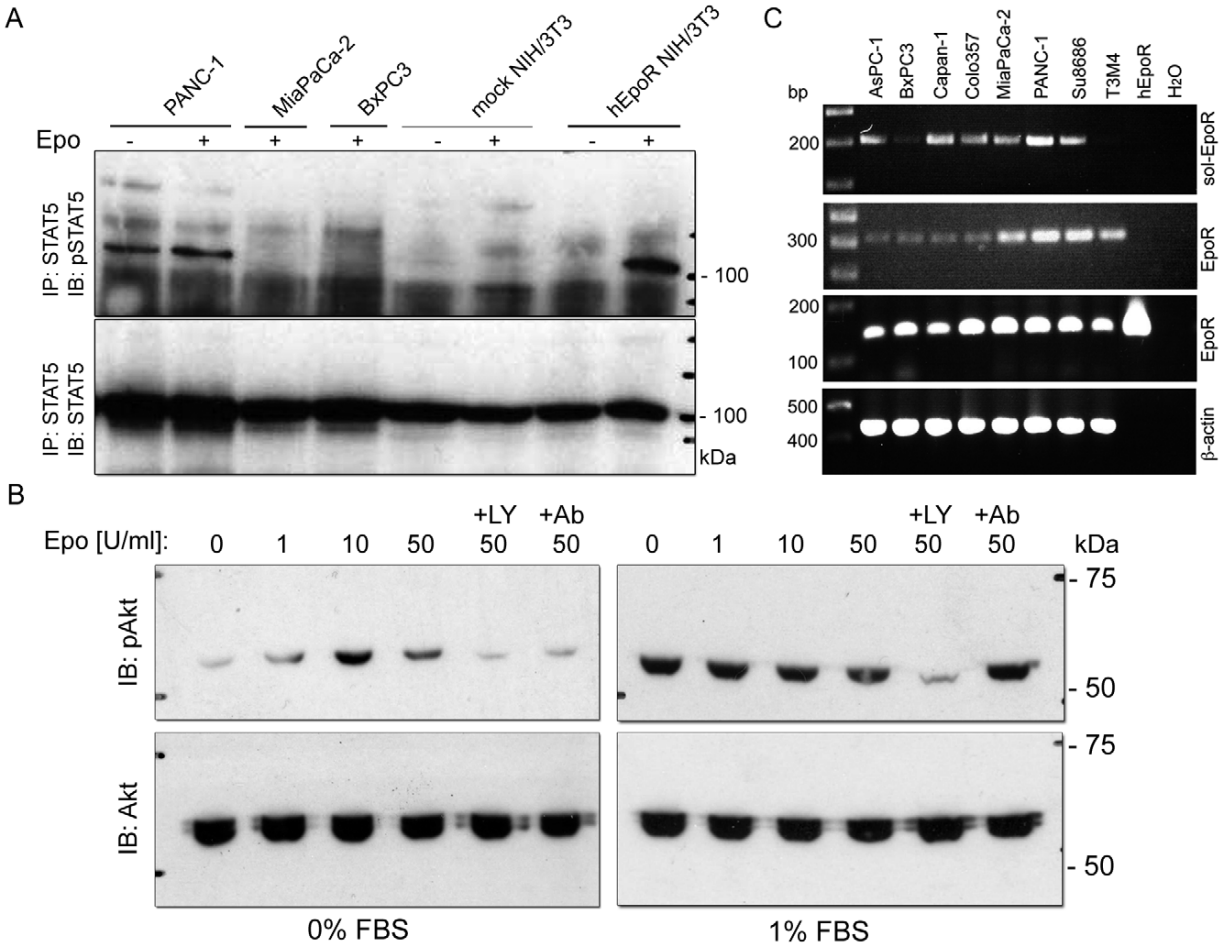
Activation of PI3K/Akt but not Jak2/STAT5 signaling in PDAC cells exposed to Epo. (A) Phosphorylation status of Stat5 was assessed in serum-starved pancreatic tumor cells and hEpoR-transduced NIH/3T3 cells stimulated with 50 U/ml erythropoietin (Epo) for 10 min. Clear accumulation of pSTAT5 was observed in hEpoR-NIH/3T3 but not in mock-transduced NIH/3T3 cells or in PDAC cells, independent of the level of constitutive pSTAT5 activation. (B) Phosphorylation status of Akt was assessed in serum-starved (left panel) or non-starved (right panel) PANC-1 cells consequently stimulated with Epo at 0–50 U/ml for 15 min. Epo-enhanced pAkt phosporylation was detected only under conditions of serum starvation and could be specifically inhibited by anti-EpoR antibody (+Ab) or phosphatidylinositol-3-OH kinase (PI3K) inhibitor LY2940020 (+LY). (C) Accumulation of mRNA coding for soluble EpoR isoform (upper panel; primers: EpoR_S5/6, table 2) as compared to mRNAs coding for full-length isoforms (two middle panels) and further related to expression of ß-actin (lower panel). 3′-end-based detection of EpoR mRNA was performed with antisense primers binding after (EpoR_FL1/2) or prior to a stop codon (EpoR_FL3/4) as visualized by a hEpoR plasmid carrying only the coding sequence for a full-length isoform.

Two principal signaling pathways activated upon binding of Epo to EpoR are the Jak2/STAT5 and PI3K/Akt phosphorylation cascades. Upon stimulation of serum-starved cells with 50 U/ml of Epo, phosphorylation of STAT5 was observed in hEpoR-transduced NIH/3T3 (Figure 6A) and Ba/F3 cells (not shown), but in none of three studied pancreatic tumor cell lines. Epo was unable to either initiate STAT5 activation in MiaPaCa-2 and BxPC3 cells, or to potentiate STAT5 phosphorylation in PANC-1 cells which displayed strong constitutive phosphorylation of STAT5 despite prolonged 24 h serum starvation (Figure 6A). In contrast, dose-dependent phosphorylation of Akt was observed in serum-starved PANC-1 cells showing a very weak level of constitutive Akt activation (Figure 6B, left). This induction could be blocked by the anti-EpoR antibody MAB307 or by the specific PI3K/Akt pathway-inhibitor LY294002. Potentiation of Akt signaling in primed (1% serum) PANC-1 cells was not possible (Figure 6B, right). Thus, the PI3K/Akt pathway but not the Jak2/STAT5 pathway was able to transmit the Epo signal in non-primed PDAC cells.

### Expression of the soluble EpoR- isoform in PDAC cells and lack of the functional responses to rhEPO

The behavior of PDAC cells in an Epo-rich environment (0–50 U/ml) has been studied by assessing the proliferative, invasive and survival potential of three PDAC cell lines (PANC-1, MiaPaCa-2, BxPC3) under conditions of oxygen deprivation, serum starvation and chemotherapeutic treatment (5-fluorouracil and gemcitabine). Neither of these experiments demonstrated any statistically significant pro-malignant or pro-survival effects of exogenously added rhEpo (data not shown). Although we could not exclude the inertness of Epo towards the tumor cells under the studied conditions, we hypothesized that a soluble isoform of EpoR (sol-EpoR) might block Epo signaling by binding of Epo and thereby decreasing receptor-mediated signal transduction. Such a sol-EpoR variant is known to rapidly accumulate up to the nanogram level in the growth medium of tumor cells as soon as 1.5 hours after medium renewal [39]. Probably, sol-EpoR accumulation could occur in the long-term functional studies but not in the short-term signaling experiments. RT-PCR analysis showed varying levels of the alternatively spliced mRNA transcripts coding for the sol-EpoR isoform in all eight studied cell lines (Figure 6C). We concluded that EpoR-positive PDAC cells are equipped to respond to Epo but sol-EpoR might antagonize Epo signaling, with higher Epo having a greater chance of overcoming sol-EpoR and reaching tumor cells *in vitro* and in sEpo^high^ patients.

## Discussion

Today, the safety of Epo in the oncologic setting is still an issue of concern. Several studies have attempted to elucidate whether and how Epo might stimulate tumor progression, but the data still do not clearly explain the underlying mechanisms [14]. Although the findings of mRNA transcripts coding for multiple EpoR isoforms are abundant and convincing, the observation regarding cell surface expression, down-stream signaling and functional consequences are rather rare and controversial [23], [27]. In the present study, we investigated the effects of endogenous and exogenous Epo on PDAC progression and chemoresistance.

We showed that sEpo^high^ (≥16 mU/ml)—but not Hb—was an independent, negative prognostic survival marker in PDAC. Although the physiological Epo response seems to be impaired in PDAC, ectopic non-malignant sources of Epo such as hepatocytes and capillaries surfaced as potential candidates for restoring sEpo levels in the circulation and for creating Epo-rich niches at different locations. Since we succeeded in proving EpoR expression at the mRNA and protein level, as well as EpoR initiation of PI3K/Akt signaling in PDAC cells, a direct impact on the tumor cells emerged as a highly probable event to be associated with the negative clinical associations seen in sEpo^high^ PDAC patients. However, we could not track any effects of Epo on proliferation, invasion or chemoresistance. PI3K/Akt activation by Epo has recently been reported in other malignancies and was associated with either antiapoptotic or proliferative effects or with no impact on cellular proliferation [40]–[42]. Since Epo did not affect PDAC cells even under hostile environmental conditions (hypoxia, serum starvation), one could suggest that neither high sEpo levels creating Epo-rich niches, nor administration of rhEpo will provoke pancreatic malignancy.

The clinical impact and interpretation of these results are complex, however. First, the minimal Epo concentrations needed to demonstrate Akt-phosphorylation (10 U/ml) *in vitro* were 1000-fold higher than normal blood concentrations in humans, ranging from 5 to 34 mU/ml [18], [31], [32], although we cannot exclude higher levels of Epo accumulating near tumor cells in tissues. PDAC cells also needed to be serum-starved to unmask Epo-mediated Akt-phosphorylation, calling into question whether Epo is even able to activate Akt signaling *in vivo*. Second, the lack of functional consequences could result from the blockade of Epo-binding to EpoR by sol-EpoR secreted by tumor cells [39]. Since sol-EpoR was found in human plasma, this isoform might emerge as an important modulator of sEpo availability for normal and malignant cells, thus separating sEpo^high^ patients into a group where sEpo will have higher chances of overcoming neutralizing effects of sol-EpoR while reaching pancreatic or disseminated tumor cells. A recently published mathematical model indicated that Epo signaling is a finely tuned process in which the balance between Epo ligand and available EpoR crucially affects receptor activation and potentially downstream signaling in hematopoietic cells [20]. Thus, an array of interfering factors—variability of EpoR expression, pAkt status, and existence of the neutralizing sol-EpoR isoform—could determine the outcome of signaling in PDAC cells by disturbing the Epo/EpoR balance. As a result, Epo signaling and potential pro-malignant effects would be impeded in tumor cells of patients with a low level of sEpo, and sEPO ≥16 mU/ml could represent a level of saturation sufficient to overcome all existing limitations. This hypothesis would argue to not administer rhEpo if sEpo levels are exceeding a critical threshold. The testing of the available mathematical model in an appropriate experimental system and its validation in clinical material are further steps towards clarifying the role of the Epo/EpoR system in PDAC and towards optimization of rhEpo-based treatments of anemic cancer patients.

The association of high preoperative sEpo levels with decreased survival is in line with recent findings in endometrial, renal and non-small-cell lung cancer patients, in whom high sEpo levels were associated with poorer outcome [15], [43], [44]. However, high sEpo was also a negative prognostic factor in anemic patients with a non-malignant condition such as congestive heart failure [34]. Interestingly, increased mortality in these patients was associated with sEpo levels >22.6 mU/ml—a cut-off close to that observed in the present study (sEpo^high^ ≥16 mU/ml). Possibly, altered Epo response is a sign of systemic disease accompanying pancreatic disorders. Indeed, both hallmarks of Epo expression in PDAC— ectopic expression and distant delivery—were associated with non-malignant structures and also found in inflammatory pancreatic disease. The inflammatory reaction affecting the pancreas is known to alter a profile of secreted pro-inflammatory Interleukin (IL)-1, IL6, TNFα), pro-angiogenic (IL8, VEGF) and pro-fibrogenic (IL10, TGFβ) cytokines [35], [45]. Systemic action of released cytokines might profoundly change the vascularization, the secretion profile and the functioning of peripheral organs. In cancer, these reactions can be further affected by the tumor cells and yield paraneoplastic syndromes such as cachexia or immune dysfunction [46], [47]. Likewise, a number of factors regulating Epo expression in hepatocytes (IL6, PHD, HIF1 α) could be produced or influenced by primary or metastatic PDAC cells [33], [46], [48]–[50].

Our preliminary observation of an up-regulated Epo mRNA expression in the non-metastatic hepatic specimens (not shown) supports consideration of ectopic Epo production as a systemic syndrome. Still, despite all similarities, the mortality of CP patients is low. A number of recent studies showed preferential expression of EpoR in cancer-initiating (stem) cells [51], [52], enabling a novel explanation for the negative clinical associations of high sEpo (or administered rhEPO) through the direct effects on tumor cells. One could speculate that systemic induction of hepatic or vascular Epo alters the Epo gradient between the circulation and tissues, so provoking an egress of EpoR-positive cancer stem cells from dormant locations (bone marrow or pancreas) into the circulation and/or from the circulation to remote niches (pancreas or liver). Metastasized tumor cells might further upgrade Epo synthesis at distant locations, thus replicating the chain of events and scaling-up metastatic spread. Consequently, the adverse outcome of Epo^high^ patients could be related to the higher odds of reaching certain Epo thresholds, which are necessary for the mobilization or recruitment of EpoR-positive cancer (stem) cells, notwithstanding sol-EpoR. If ectopic Epo response is inherently capped, patients having a higher Epo response as a result of systemic disease would be pre-disposed to early and faster development of metastasis, thus explaining how the sEPO^high^ can be an independent prognostic marker *and* linked to metastatic status.

In conclusion, the present study demonstrated that PDAC cells are equipped to respond to Epo through functional EpoRs, although conditional activation of PI3K/Akt signaling can possibly be offset by the soluble isoform of EpoR. Despite an impairment of the Epo-response in PDAC patients, sEpo levels could be restored by ectopic production in capillaries and hepatocytes. Elevation of sEPO beyond 16 mU/ml was associated with worse survival independent of metastatic status. Yet higher levels of sEPO could be observed in stage IV patients. Further trials are required to clarify whether high Epo predisposes to or results from metastatic progression and whether the suspected mobilization/recruitment of EpoR-positive cancer-initiating cells is the likely cause of pro-malignant effects for adverse outcome in rhEPO-treated oncologic patients. While high serum Epo levels might be an inherited factor increasing the probability that Epo overcomes the antagonizing effects of sol-EpoR in PDAC patients, elucidation of the threshold determining the outcome of the Epo/EpoR interplay by using mathematical models might help to optimize Epo-based treatments.
